# Healthcare Providers' Intention to Use Technology to Attend to Clients in Cape Coast Teaching Hospital, Ghana

**DOI:** 10.1155/2021/5547544

**Published:** 2021-11-05

**Authors:** Richard Okyere Boadu, Mary Adama Lamptey, Kwame Adu Okyere Boadu, Godwin Adzakpah, Nathan Kumasenu Mensah

**Affiliations:** ^1^Department of Health Information Management School of Allied Health Sciences, College of Health and Allied Health Sciences, University of Cape Coast, Cape Coast, Ghana; ^2^Health Information Management Unit, Ussher Polyclinic, Ghana Health Service, Accra, Ghana; ^3^School of Medicine and Dentistry, College of Health Sciences, Kwame Nkrumah University of Science and Technology, Kumasi, Ghana

## Abstract

**Background:**

Patient records' relevance is associated with a variety of needs and objectives. Substantiating the health of patients perpetually and allowing professionals in the medical field to assess both signs and symptoms that fall in a relatively wider temporal point of view and contributions that lead to enhanced diagnoses and treatment are all quintessential of patient records. The advancement of information technology systems has led to the anticipation that development will be put into digitization and electronic means of storing patient records in order to grease their handling. Cape Coast Teaching Hospital (CCTH) is piloting implementation of patient's electronic health record system. The introduction of the electronic health record system known as Lightwave Hospital Information Management System (LHIMS) was to provide a permanent solution to patients' continuity of care. User's acceptance of new information technology is seen to be one of the most challenging issues in information system. This study assesses healthcare providers' (HP') behavioural intention to use LHIMS to attend to clients in Cape Coast Teaching Hospital and other factors influencing it.

**Methods:**

A nonexperimental cross-sectional study was used to obtain information from 84 HP recruited from the various departments and units in CCTH who use LHIMS to attend to clients. The sample size of 90, representing 8% of HP in CCTH, was randomly selected from the various departments and units. However, 84 (indicating 93.3% response rate) of the selected HP were available during the period of the research.

**Results:**

Perceived ease of use (PEOU) of LHIMS had the strongest direct effect on perceived usefulness (PU), with a highly significant path coefficient of 0.75. PU had the greatest impact on attitude about HP' behavioural intention to use (BIU) LHIMS to attend to patients' healthcare delivery in CCTH (0.91). This relationship was highly significant at *p* < 0.001. PEOU did not have a significant direct effect on attitude about LHIMS use, as hypothesized in the original technology acceptance model. However, attitude towards use had a strong significant effect on HP' BIU of LHIMS, with a strong statistically significant path coefficient of 0.98 at *p* < 0.001.

**Conclusions:**

We conclude that attitude towards use have a significant influence on HP' behavioural intention to use LHIMS to attend to clients in Cape Coast Teaching Hospital.

## 1. Introduction

Patient records' relevance is associated with a variety of needs and objectives. Substantiating the health of patients perpetually and allowing professionals in the medical field to assess both signs and symptoms that fall in a relatively wider temporal point of view and contributions that lead to enhanced diagnoses and treatment are all quintessential of patient records. The importance of patient record is also well appreciated in matters relating to legalities. In as much as it can be used in trials, it paves way for doubts to be cleared; hence, behaviours are appreciated, and in the long run, patients, medical personnel, and all engaged parties are safeguarded. Furthermore, these records have information that depicts the progression of patients. This information helps in evaluating procedures and the aftermath of these methodologies. Ultimately, they are used in research. Large hospitals have challenges with storage space for these conventional records (printed records). Due to the heightened call for space, some of these records are left in shambles. It is quite difficult to either keep or access information from them. Also, most of these documents are incoherent or incomplete [1–5].

Retrospective and epidemiological analyses are held back because of the adverse effects of incoherent information and storage challenges that make a great number of records inaccessible; research is compromised as a result. It was no surprise when Santos and colleagues [6], professed that classic information system is seen “as a limited vehicle of communication that has been surpassed by modern digital technology” [6, 7]. The advancement of information technology systems (generically denoted as IT) has led to the anticipation that development will be put into digitization and electronic means of storing patient records in order to grease their handling. In as much as policy analysts and policy makers had appreciated the potential use of IT in the healthcare, the momentum was not the same as observed in other sectors. Hospitals that use electronic records are not many [2, 8–11], a situation that is similar to Ghana's health system.

CCTH is one of the first tertiary healthcare facilities which was used as a pilot site to implement patient's electronic health record in Ghana. The facility had been using paper records since it was established. The hospital's client attendance has experienced an increasing trend over the years because it serves as a referral facility for the Central, Western, and Western North regions of Ghana. Due to the increasing attendance, the facility was facing challenges such as storage space for patients' records, issues with missing of patient records which did not promote continuity of care. Other challenges include delays in retrieving of patient records which contributed to long patient waiting time, inconsistent network, inadequate training for unskilled record staffs, loss of patients' supplementary records, and high cost of procuring patients' folders, among others. The introduction of the electronic health record system known as LHIMS in December, 2017 and fully rolled out in 1st April, 2018 was to provide a permanent solution to some of the above challenges of the paper record system. LHIMS provides accurate, up-to-date, and complete information about patients at the point of care, reduces cost, enabling quick access to patient records for more coordinated and efficient care, provides more efficiently diagnose patients, reduces medical care errors, and provides safe care, promote legible, complete documentation, and accurate streamlined coding and billing. Notwithstanding the positive impact LHIMS is having on the healthcare delivery in the facility, it also has some challenges such as interruption of power supply and unreliable internet connectivity which slows down workflow. The advantage or otherwise of any system can only be known when the users of the system accept and use it. User's acceptance of new information technology is seen to be one of the most challenging issues in information system of which healthcare professionals are no of exception.

Improving the intention and skill of using computers should be a major point of attention for teaching hospitals who wish to improve their care providers' attitudes to remote monitoring and willingness in using information technologies. Besides, the awareness of professionals is crucial for improving willingness [12]. Undoubtedly, researchers investigated and published a theoretical model to elucidate user's information technology acceptance and use, for example, the theory of reasoned action [13, 14], the theory of planned behaviour [15, 16], besides the technology acceptance model (TAM) [17, 18], and customer adoption of information technology in the direction of a unified view model [18]. Among those theories and models, “one of the commonly applied models of information technologies acceptance and engagement is the TAM” [19]. TAM was applied to explain the customer acceptance of innovation utilized in a diverse environment setting. With the upsurge in technological necessity in our lives and world economy connection, several researchers linked with the scholarly community and industry have been enthusiastically concerned with studying end-user adoption intentions of diverse technologies [20]. Zhou et al. conducted a study about the contributing factors to nurses' behavioural intention to use Hospital Information Technologies (HITs) based on the model of “Unified Theory of Acceptance and Use of Technology.” They (Zhou et al.) presented “a piece of empirical evidence for hospital administrators in developing countries especially Ghana, to assess the success probability of new HITs before and after their implementation” [21].

This study assesses HP' behavioural intention to use LHIMS to attend to clients in CCTH and other factors influencing it.

## 2. Methods

### 2.1. Study Design

We used nonexperimental cross-sectional study which employed quantitative technique to obtain information from 84 HP selected from the various departments and units in Cape Coast Teaching Hospital who use LHIMS to attend to clients. Respondents were studied at a particular point in time, and the results were analyzed and presented soon after.

### 2.2. Profile of Study Area

This study was carried out at Cape Coast Teaching Hospital in Cape Coast Metropolis of the Central Region of Ghana. The Cape Coast Teaching Hospital is one of the agencies under the Ministry of Health. With a current bed capacity of 400, the hospital is mandated to provide tertiary clinical services, serve as a training for graduate and postgraduate medical programs and to undertake research into emerging health problems. It also serves as the referral facility for the health facilities in the Central, Western, and Western North regions of Ghana. It was established in August, 1998 as the Central Regional Hospital and later upgraded to a Teaching Hospital status in March, 2014, following the establishment of the School of Medical Science at the University of Cape Coast, Ghana. Cape Coast Teaching Hospital is also accredited postgraduate training by the Ghana College of Physicians and Surgeons. The hospital is the main training centre for students of the School of Medical Sciences of the University of Cape Coast. It also collaborates with other schools and colleges including School of Nursing and Midwifery as well as School of Health and Allied Sciences. These schools train students at both undergraduate and postgraduate levels. The hospital is geographically located at the northern part of Cape Coast and bounded on the North by Abura Township, on the South by Pedu Estate and 4^th^ Ridge, Nkanfoa on the East, and Abura/Pedu Estate on the West.

### 2.3. Study Population

The study population involves all HP in the various departments and units of CCTH who use LHIMS attend to clients. The sample size of 90 representing close to eight percent of HP in CCTH was randomly selected from the various departments and units. However, 84 (indicating 93.3% response rate) of the selected HP were available during the period of the research. A simple random sampling was used to select HP who use LHIMS, from the various departments and units, into the study. In order to give equal opportunity to all eligible staff to participate in the study, a simple random sampling was used to select participants. At each department/unit a list of staff with serial ID was generated in a consecutive order (e.g., 001, 002,…, *N*), where *N* is the total number of staffs who were engaged in LHIMS process. A random generator (Mobile App) was used to select participants based on their serial ID until the study population was covered. Staffs who were selected and voluntarily consented to participate were included in the study.

### 2.4. Measurements and Data Analysis

A key measurement issue of the study concerns the multidimensional nature of most of the technology acceptance model (TAM) depicted in the conceptual framework. Most of the inputs of TAM are measured through series of continuous or Likert scale indicators which are used to generate indices following Davis Conceptual Research Model [17]. The questions were based on prior studies with modifications to fit the specific context of the LHIMS usage and subsequently developed from the TAM scales adapted [17, 22, 23]. All the constructs in the research model were operationalized using standard scales from past literature. Our research TAM model consisted of 17 items that measured “perceived usefulness” (4 items), “perceived ease-of use” (4 items), “attitude towards usage” (5 items), and “behavioural intention to use LHIMS” (4 items). The response scale for all items was a seven-point, positively packed Likert scale [24, 25] coded as 7: strongly agree, 6: moderately agree, 5: slightly agree, 4: neutral, 3: slightly disagree, 2: moderately disagree, and 1: strongly disagree. Stata SE version 15 was used to produce descriptive analysis of categorical variables using proportions. Statistical reliability test of the variables in the dataset was assessed using Cronbach's alpha reliability coefficient. This method was applied to assess the internal consistency of the survey items [25, 26]. We also conducted goodness-of-fit indices for scale validity tests using chi-square statistic. The chi-square test of statistic is a natural index used to measure the goodness-of-fit between data and the model [27, 28]. A model fit index test using goodness of fit was also determined to know whether the model has the capacity to predict how accurate the model fits the set of observations [25, 28]. Structural equation modelling (SEM) technique was employed to evaluate the direct and indirect effects of the independent variables on the health staffs' behavioural intention to use (BIU) LHIMS in the model. Stata SE version 15 was used to calculate the path coefficients and test the model hypothesis. A *p* value of less than 0.05 served as basis for considering a statistically significant model.

### 2.5. Research Model and Hypotheses

The technology acceptance model (TAM) is used in this study for its predictive ability in studies involving students [23, 29–32]. The causal relationships between perceived usefulness (PU), perceived ease of use (PEOU), attitude towards usage (ATU), and behavioural intention to use (BIU) technology are specified in the TAM to reflect the new environment of LHIMS. PU is defined as the degree to which an individual believes that using LHIMS would enhance his or her performance in health care delivery, whereas PEOU refers to the degree to which an individual believes that using the system would be free of cognitive effort. TAM suggests that actual usage of the system is determined by the users' behavioural intention to use (BIU) the system, which is determined by users' attitude towards using the system and their perceived usefulness and ease of use of the system [17]. Together, PU and PEOU constitute a significant influence on ATU, which in turn affects the BIU. In addition, PEOU has also been shown to significantly influence PU [23, 32].

Similarly, the BIU system is posited to be affected by ATU. In accordance with the research objective and consistent with the related literature, this study tested the following hypotheses:

*H1*: PU will have a significant influence on ATU.

*H2*: PEOU will have a significant influence on ATU.

*H3*: PEOU will have a significant influence on PU.

*H4*: ATU will have a significant influence on healthcare providers' BIU the LHIMS.

**Figure 1:**
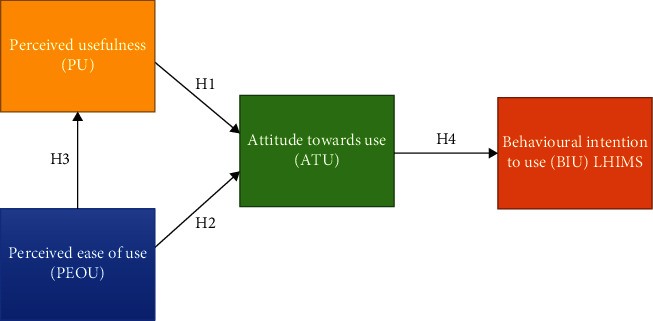
Conceptual research model [17].

These hypotheses give rise to the research model (Figure 1) represented as a causal relationship schema and used as a point of departure for this research. The boxes represent the constructs which were measured by a set of items, with arrows representing hypotheses 1 to 4.

### 2.6. Ethical Considerations

This study is noninvasive one and did not cause any physical harm. To deal with ethical issues, approval was sought and granted by the CCTH Ethical Review Committee. An introductory letter was sent to the Chief Executive Officer of the hospital to seek permission to begin the fieldwork. At each interview point, there was self-introduction by the investigator to the respondents and the purpose of the study was comprehensively explained to them. Respondents were given the opportunity to decide whether to partake in the study. The respondents were assured of the confidentiality of their identity in the study.

### 2.7. Reliability and Validity

The questionnaire for the study was pretested at Cape Coast Metro Hospital, which is not part of the study area but has common characteristics with Cape Coast Teaching Hospital. After the pretesting, some questions were rephrased to reflect reality on the ground.

## 3. Results

The gender distribution of the participants comprises of majority being (62%) females and the rest (38.1%) males. Of the 84 participants, 4 out of 10 were from 21 to 30 years. Fifteen percent of respondents were from 31 to 40 years, and only 1% was from 41 to 50 years. Remarkably, about 43% of respondents did not disclose their age. In summary, the mean age was 29.7, range was 21-63, variance was 18.8, and the standard deviation was 4.3. In terms of educational background, majority of the participants (64%) were professional diploma or degree holders. Those who had completed a bachelor program accounted for 19 percent while their master counterparts were close to 11 percent. Participants with postsecondary school and other education were about two percent each. Those in secondary were around one percent. Noticeably, participants with less working experience (<4 years) are more than half of the total participants interviewed. Particularly, 25 percent of the participants had worked for three years, and those who had worked for a year were 21 percent. Nearly 11 percent of participants had worked for two years whereas closed to 42 percent had worked for 4 years or more. Of the 84 participants who took part in this survey, 43 percent were nurses. Midwives constituted about 23 percent while medical officers constitute 19 percent. The rest consist of pharmacists, public health nurses, health information officers, medical assistants and biostatisticians, laboratory technicians, and other facility staff.

### 3.1. HP' Perceived Usefulness of LHIMS

“Perceived usefulness” scale incorporated four dimensions: did the use of LHIMS by respondents enhance effectiveness in healthcare delivery, increase productivity at work, and enable the accomplishment of tasks quickly? Was it useful? In general, over 7 out of 10 respondents perceived LHIMS to be useful. With the indices that were used to measure “perceived usefulness,” 76.5 percent of respondents perceived that LHIMS improved their effectiveness in healthcare delivery. While three-fourth of respondents believed LHIMS increased their productivity at work, 69 percent were able to accomplish tasks more quickly with LHIMS, and 86.4 percent found the use of LHIMS useful. The overall perceived usefulness of LHIMS was 76.5 percent (Table 1).

### 3.2. HP' Perceived Ease of Use of LHIMS

Table 2 discusses the scale of index of “perceived ease of use”. Was learning to use the LHIMS interface easy for respondents? Was it clear and understandable? Was it flexible to interact with? Did the participants become skillful at usage? About 83 percent of respondents perceived ease in either learning to use the interface or understanding it with clarity. More than 8 out of 10 persons believed it was easy for them being skillful while 85 percent found the interface to be flexible to interact with. The overall perceived ease of use was 84 percent (Table 2).

### 3.3. HP' Attitude towards LHIMS Usage

In analyzing respondents' “attitude towards usage” of the LHIMS system, these factors were considered: whether respondents had a general favourable attitude towards using the LHIMS system, did respondents believe it was a good idea to use the LHIMS for healthcare delivery, did the use of LHIMS provide much enjoyment, expectations of LHIMS in the future, and the overall enjoyment of the system. The overall attitude towards use was an overwhelmingly close to 83 percent of respondents. While 9 out of 10 respondents binged on the idea of using LHIMS for healthcare delivery, 85.2 percent either expected their use of LHIMS to continue in the future or generally enjoyed using the LHIMS. About 8 out of 10 respondents believed they had a generally favourable attitude towards use. Interestingly, 30 percent of respondents did not enjoy using the LHIMS (Table 3).

### 3.4. HP' Behavioural Intention to Use LHIMS

For “behavioural intention towards use”, respondents' intention to use LHIMS during encounter with patients, intention to use LHIMS as often as possible, and intentions to use LHIMS in the future were considered as described in Table 4. While the overall behavioural intention to use was 85 percent, a whopping 89 percent intended to use the LHIMS often. About 85 percent of respondents planned to use LHIMS in the future while close to 83 percent intended to use it when they encounter patients (Table 4).

### 3.5. Reliability of Research Constructs

Cronbach's alpha reliability coefficients were calculated using Stata version 15 to determine the internal consistency of all study constructs (latent endogenous variable scales). Our results show excellent scale reliability for all constructs (Table 5). Hair and colleagues [25] indicate that reliability should be greater than 0.70.

Root mean squared error of approximation < 0.08 indicates good fit.

### 3.6. Structural Equation Modelling (SEM) Analyses

Table 6 shows individual parameter estimates of HP' behavioural intention to use of LHIMS to attend to patients' healthcare delivery in Cape Coast Teaching Hospital: direct/indirect/total effects.

### 3.7. Direct Effect

The variable estimates test the statistical significance and power of each hypothesized path in the model. The exogenous (independent) factors may directly or indirectly affect the endogenous (dependent) variables. Standardized estimates allow the author to evaluate the relative contribution of predictor variable to each outcome variable, as well as compare across groups [33].  Table 7 shows standardized direct and indirect effects of the independent variables on the dependent variables.

PEOU had the strongest direct effect on PU, with a highly significant path coefficient of 0.75. PU had the greatest impact on attitude about HP' behavioural intention to use LHIMS to attend to patients' healthcare delivery in Cape Coast Teaching Hospital usage (0.91). This relationship was highly significant at the 0.001 level. PEOU did not have a significant direct effect on attitude about LHIMS use, as hypothesized in the original technology acceptance model. However, attitude towards use had a strong significant effect on HP' BIU of LHIMS, with a strong statistically significant path coefficient of 0.98 at *p* < 0.001.

**Figure 2:**
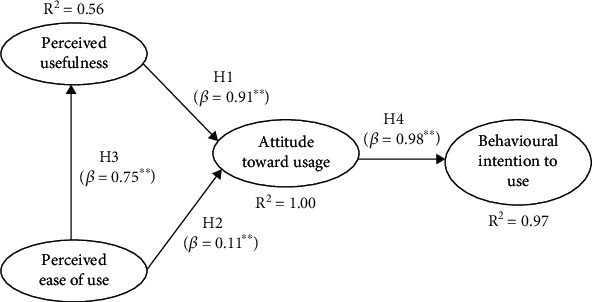
SEM path diagram of latent variables. LR *χ*^2^ (100) = 329.16; *p* < 0.0001. Overall, *R*^2^ = 1.00. Statistically significant: ^∗^*p* < 0.05; ^∗∗^*p* < 0.001.

Figure 2 shows *R*^2^ values for each of the dependent parameters. PEOU accounted for 56% of the variation in PU. The combinations PEOU and PU also accounted for 100% variation in ATU of LHIMS. The overall model shows 97% of the variation of HP' BIU of LHIMS as captured by the independent variables in the model (Table 6).

### 3.8. Indirect Effect

Standardized indirect effects are summarized in Table 6 to throw more light on the indirect effect of each of the independent variable on the dependent variable. PEOU had indirect effects on ATU, which were mediated by PU. PU and PEOU had indirect effects on HP' BIU of LHIMS, all of which were mediated by ATU. Indirect effects are in addition to any direct effects the exogenous variables may have on endogenous variables.

### 3.9. Total Effects

All independent variables in the model had a strong statistically significant effect on HP' BIU of LHIMS. ATU had the strongest effect (0.98), with PU (0.90) and PEOU (0.78) making noteworthy contributions (Table 6).

## 4. Discussions

### 4.1. HP' Perceived Usefulness of LHIMS

New pieces of application software are created regularly to suit the needs of users. Nonetheless, many people are conservative and not subject to use new software. The educated and technology-driven mass will be the best people to test new technology since they actually embrace new technology in order not to be archaic. No matter how interesting the new technology will appear to these people, lack of electronic content in their preferred language, the rapid build-up of the tools to access new technology, and high cost of new software will prevent people from getting the software [34–36]. On the contrary, majority of LHIMS users had a positive perception with regard to ease in usage with both friendly interface and easy instructions with clarity. They (respondents) believed it was easy for them being skillful, and they also found the interface to be flexible to interact with. In all, 3 out of 4 HP perceived LHIMS as a very useful electronic patients' record management system.

### 4.2. HP' Behaviour (Attitude, Frequency, Intensiveness, and Intention) towards LHIMS Usage

Attitude, frequency, intensiveness, and intention determine how a user will use a new application software; hence, users' behaviour towards a new technology is observed. In 2012, Nair and Das found out that users' behaviour towards using a new technology is affected by how they projected and how easy it would be to use the new software [37]. Nonetheless, it is believed that enough training will make users more comfortable in using a new application software, therefore improve their positive attitude towards usage. This study demonstrated that an overwhelming number of HP had a favourable attitude towards using the LHIMS. For instance, nine out of ten HP binged on the idea of using LHIMS for healthcare delivery whereas over 8 out of 10 HP either expected their use of LHIMS to continue in the future or generally enjoyed using the LHIMS. This outstanding performance may be attributed to HP' perception of ease of LHIMS usage. Perceived usefulness is hypothesized to affect behavioural intention directly. Therefore, people generally have a great attitude towards a new technology when they think it is easy to use it [34, 38–40].

### 4.3. Relationship between HP' Perceived Usefulness and Their Attitude towards Usage of LHIMS

HP' attitudes towards a system would be incomplete without a discussion on the technology acceptance model [41, 42]. TAM focuses on factors that determine the HP' behavioural intentions towards accepting a new technology. It assumed that the intention to use a system is influenced by individual's attitude towards using the system [38, 40]. Our study approbated this relationship, as attitude towards use of LHIMS had a strong significant effect on HP' BIU of LHIMS with a strong statistically significant path coefficient of 0.98 at *p* < 0.001. Also, an individual's attitude is influenced by how useful they think the new technology would be. If they perceive it to be very useful, they are motivated towards getting the best out of the new technology. TAM also found that perceived usefulness is a crucial determinant of attitude towards using a new technology [38, 40]. In addition to this, our study confirms this assertion. For instance, PU had the greatest impact on attitude about HP' behavioural intention to use LHIMS to attend to patients' healthcare delivery in Cape Coast Teaching Hospital. Usage had a coefficient of 0.91 and was highly significant at the 0.001. It is important to note, however, that usage of a technology may not be voluntary, and the users had no choice but to adapt to the technology because of how useful it appeared to be [43–45].

### 4.4. Relationship between HP' Perceived Ease of Use of LHIMS and Their Attitude on Usage

Attitude towards use is the “degree to which an individual evaluates and associates the target system with their job.” In TAM, attitude towards usage is referred to as the appraised effect of positive or negative feeling of individuals in showing a particular behaviour. Perceived ease of use (PEOU) will have a significant influence on attitude towards usage [37, 46–48]. When people (staff) perceive the system (LHIMS) as one that is easy to use and fairly free of mental effort, they may have a favourably positive attitude towards the use of the system [46]. Users have their own perceptions towards a new technology. Their attitude towards using the technology is based on how easy they think it would be for them to use it. Perceived ease of use is the degree to which a person believes that using a particular system is effort-free [49]. TAM assumed that one's intention to use a system can be induced by the individual's attitude towards using the system. If the user perceives the system to be easy to use, he will have an agreeable intention to use it [38]. Antithesis to our study, PEOU did not have any significant effect on attitude towards LHIMS use, as hypothesized in the original TAM. Nonetheless, other studies stated that subjective norms and perceived usefulness as well as one's educational level may determine behavioural intention to use the system [49–51].

### 4.5. Relationship between HP' Attitude towards Usage and Their Behavioural Intention to Use LHIMS

People's attitude is influenced by how useful they think the new technology is. If they consider it to be very useful, they exhibit a good attitude in using the new technology. An explanation might be that when users perceive a system as one that is easy to use and nearly free of mental effort, they may have a favourable attitude towards the usefulness of the system. Therefore, the behaviours towards use would be auspicious [46, 48, 52–54]. This highlights the fact that perceived usefulness will inspire users to have a positive attitude towards use. Analogous to the other studies, our study suggested that attitude towards use of LHIMS had a strong significant effect on HP' BIU of LHIMS, with a strong statistically significant path coefficient of 0.98 at *p* < 0.001. Nevertheless, performance expectancy, effort expectancy, and facilitating conditions are the factors that significantly affect a user's intentions and behaviours towards using a new technology [38], underlying the fact that attitude and behavioural intentions correlate. Basically, it is the perceived usefulness and ease of use that affect the behavioural and attitudinal intentions towards use.

### 4.6. Strength and Limitations of the Study

The TAM model is theoretical. External variables like PU and PEOU have a direct impact on HP' intent to use the new technology like LHIMS. Evaluating behavioural intent is subjective. The belief that users will have a good ATU once they perceive a system to be easy to use is flawed. These perceptions may probably be from HP' supervisors who may enforce that they use LHIMS regardless of their discernment on the technology. In this study, TAM could not give any in-depth antecedent to behavioural influence to LHIMS usage and was not robust to elucidate user's behaviours on accepting LHIMS. The main strength of this research is the use of structural equation modelling (SEM) for the analysis. The application of the SEM has several advantages compared to multiple regression method in the sense that we are able to determine the extent of effect of the independent variable on the dependent variable. SEM enables us to show both the direct and indirect effects of the independent variables on the dependent variable as compared to multiple regression method. An important limitation of the study is small sample involved in the study making it difficult to draw a general conclusion for the entire country. This is as a result of the targeted users of the LHIMS at the Cape Coast Teaching Hospital in the Central Region of Ghana.

## 5. Conclusions

All independent variables in the model of our study (Figure 2) had a strong statistically significant effect on HP' BIU of LHIMS. ATU had the strongest effect, with PU and PEOU making remarkable contributions. It is believed that that PEOU had a significant influence on ATU of LHIMS. The success of LHIMS can be determined by HP' acceptance of the system, measured by three factors: PU, PEOU, and ATU of the system, and these three move hand in hand. BIU, in turn, is determined by these belief factors: PU and PEOU. When HP have a positive attitude towards using LHIMS, they are likely to use the system frequently and intensively and may have a favourable intention towards using the system. We conclude that HP have positive behavioural intention to use LHIMS to attend to clients in Cape Coast Teaching Hospital.

## Figures and Tables

**Table 1:** Perceived usefulness (PU).

Indicator	Responses	
	Disagree (%)	Agree (%)
Using the LHIMS enhanced my effectiveness in healthcare delivery	19 (23.5)	62 (76.5)
Using the LHIMS increased my productivity in my work	20 (24.7)	61 (75.3)
Using the LHIMS enabled me to accomplish tasks more quickly	25 (30.9)	56 (69.1)
I found using the LHIMS useful	11 (13.6)	70 (86.4)
Overall perceived usefulness	19 (23.5)	62 (76.5)

Source: 2020 Survey.

**Table 2:** Perceived ease of use (PEOU).

Indicator	Responses	
	Disagree (%)	Agree (%)
Learning to use the LHIMS interface was easy for me	14 (17.3)	67 (82.7)
The LHIMS user interface was clear and understandable	14 (17.3)	67 (82.7)
It was easy for me to become skillful at using the LHIMS interface	13 (16.0)	68 (84.0)
I found the LHIMS interface to be flexible to interact with	12 (14.8)	69 (85.2)
Overall perceived ease of use	13 (16.0)	68 (84.0)

Source: 2020 Survey.

**Table 3:** Attitude towards usage (ATU).

Indicator	Responses	
	Disagree (%)	Agree (%)
I have a generally favourable attitude towards using LHIMS system	15 (18.5)	66 (81.5)
I believe it is a good idea to use LHIMS for healthcare delivery	7 (8.6)	74 (91.4)
Using the LHIMS system provided me with a lot of enjoyment	25 (30.9)	56 (69.1)
I expect my use of LHIMS to continue in the future	12 (14.8)	69 (85.2)
Overall, I enjoyed using the LHIMS	12 (14.8)	69 (85.2)
Overall attitude towards usage	14 (17.5)	67 (82.5)

Source: 2020 Survey.

**Table 4:** Behavioural intention to use (BIU).

Indicator	Responses	
	Disagree (%)	Agree (%)
I intend to use LHIMS during my encounter with patient/client	14 (17.3)	67 (82.7)
I intend to use the LHIMS as often as possible	9 (11.1)	72 (88.9)
I plan to use the LHIMS in the future	12 (14.8)	69 (85.2)
Overall behavioural intention to use	12 (14.8)	69 (85.2)

Source: 2020 Survey.

**Table 5:** Reliability of research constructs.

Scale	Number of items	Reliability
Perceived usefulness (PU)	4	0.8433
Perceived ease of use (PEOU)	4	0.8247
Attitude towards usage (ATU)	5	0.7769
Behavioural intention to use (BIU)	4	0.7672

Source: 2020 Survey.

**Table 6:** Individual parameter estimates: direct/indirect/total effects.

Causal path	Standardized direct effects		Standardized indirect effects		Standardized Total effects	
	Path coefficient	*p* value	Path coefficient	*p* value	Path coefficient	*p* value
PEOU⟶PU	0.7502	*p* < 001^∗∗^	No path		0.7502	*p* < 001^∗∗^
PU⟶ATU	0.9131	*p* < 001^∗∗^	No path		0.9131	*p* < 001^∗∗^
PEOU⟶ATU	0.1121	0.427	0.6851	*p* < 001^∗∗^	0.9131	*p* < 001^∗∗^
ATU⟶BIU	0.9824	*p* < 001^∗∗^	No path		0.9824	*p* < 001^∗∗^
PU⟶BIU	No path		0.897	*p* < 001^∗∗^	0.897	*p* < 001^∗∗^
PEOU⟶BIU	No path		0.7831	*p* < 001^∗∗^	0.7831	*p* < 001^∗∗^

Statistical significance: ^∗^*p* < 0.05; ^∗∗^*p* < 0.001.

**Table 7:** Fit indices for scale validity tests of HP' behavioural intention to use of LHIMS to attend to patients' healthcare delivery in Cape Coast Teaching Hospital: direct/indirect/total effects.

Description	Value	*p* value
Model vs. saturated	*χ* ^2^ (100) = 329.158	*p* < 0.001
Root mean squared error of approximation	0.165	*p* < 0.001

Source: 2020 Survey.

## Data Availability

Data are available from the researchers. Survey data supporting this findings is restricted by Cape Coast Teaching Hospital IRB in order to protect confidentiality of the participants.

## Supplementary Materials

Supplementary Materials LHIMS data collection tool was used to assess health staffs' behavioural intention to use LHIMS to attend to clients in CCTH and other factors influencing it.
