# Supplementary material for: Healthcare Providers' Intention to Use Technology to Attend to Clients in Cape Coast Teaching Hospital, Ghana
Source: Biomed Res Int. 2021 Nov 5;2021:5547544. doi: 10.1155/2021/5547544 (PMC8589479; doi:10.1155/2021/5547544)
Supplement: Supplementary Materials — LHIMS data collection tool was used to assess health staffs' behavioural intention to use LHIMS to attend to clients in CCTH and other factors influencing it. [file 5547544.f1.pdf]

UNIVERAITY OF CAPE COAST
COLLEGE OF HEALTH AND ALLIED SCIENCES
SCHOOL OF ALLIED HEALTH SCIENCES

DEPARTMENT OF HEALTH INFORMATION MANAGEMENT

Assessment of Health Staffs' behavioural intention to use Lightwave Hospital Information Management System to attend to clients in Cape Coast Teaching Hospital

QUESTIONNAIRES FOR HEALTH STAFF

Introduction

Good morning/afternoon. I am with the Department of Health Information Management, UCC. I will be conducting interviews with staff like you in CCTH to find out your views and ideas about "**Health Staffs' behavioural intention to use Lightwave Hospital Information Management System (LHIMS) to attend to clients in Cape Coast Teaching Hospital".** Your opinion is highly essential at the same time vital as it will help us to improve the kind of service we provide to our patients. Whatever you say will be treated confidential, so feel at ease to express your candid opinion**.** Be assured that your responses will not in any way be linked to your identity. You are kindly requested to answer the questions below by indicating a tick or writing the appropriate answer when needed.

You are a volunteer. You can choose not to take part and if you join, you may quit at any time. There will be no penalty if you decide to quit the study.

THANK YOU

**Survey Variables and Codes**			
**SECTION I**			
**Que**	**Variable**	**Value**	**Code**
1	Gender	Female	1
		Male	2
2	Age (years)		
3	Education	10 years	1
		Intermediate (11-12)	2
		Bachelor (13-14)	3
		Master	4
		Professional diploma/degree (specify)--------------------	5
		Other (specify) ----------------	6
4	Working Experience (years)	1	1
		2	2
		3	3
		4+	4
5	Title of the person filling the questionnaire	Doctor	1
		Pharmacist	2
		Medical Assistant	3

Survey Variables and Codes			
		Nurse	4
		Midwife	5
		Community Nurse	6
		Public Health Nurse	7
		Nutrition Officer	8
		Disease Control Officer	9
		Health Information Officer	10
		Biostatistician	11
		Dispensary Technician	12
		Laboratory Technologist/Technician	13
		Other Facility Staff (specify) --------------------------------	14
6	Have you ever used LHIMS before in this facility?	Never	1
		Once	2
		Two to three time	3
		More than 3 times	4
7	How often have you reviewed the LHIMS?	Not at all	1
		About once each month	2
		A few times a month	3
		About once each week	4
		A few times a week	5
		Five to six times a week	6
		About once a day	7
		Several times a day	8
		Other	9
8	How often have you interacted with, or input data into the LHIMS?	Not at all	1
		About once each month	2
		A few times a month	3
		About once each week	4
		A few times a week	5
		Five to six times a week	6
		About once a day	7
		Several times a day	8
		Other	9
9	After working with the LHIMS in this facility, how experienced would you judge yourself to be?	Low experience	1
		Moderate experience	2
		High experience	3
10	With regard to technology in general, how would you describe yourself	Novice User	1
		Intermediate User	2

SECTION II

We would like to know your opinion about how strongly you agree with certain activities carried out by you. There are no right or wrong answers, but only expression of your opinion on a scale. The scale is about assessing the intensity of your belief and ranges from strongly disagree (1) to strongly agree (7). You have to determine first whether you agree or disagree with the statement. Second decide about the intensity of agreement or disagreement. If you disagree with statement then use left side of the scale and determine how much disagreement that is – strongly disagree (1), somewhat disagree (2) or disagree (3) and circle the

Survey Variables and Codes			
appropriate answer. If you are not sure of the intensity of belief or think that you neither disagree nor agree then circle 4. If you agree with the statement, then use right side of the scale and determine how much agreement that is – agree (5), somewhat agree (6) or strongly agree (7) and circle the appropriate answer. Please note that you might agree or disagree with all the statements and similarly you might not have the same intensity of agreement or disagreement and thus variations are expected in expressing your agreement or disagreement. We encourage you to express those variations in your beliefs.			
11	I have a generally favourable attitude toward using LHIMS system	Strongly Agree	7
		Moderately Agree	6
		Slightly Agree	5
		Neutral	4
		Slightly Disagree	3
		Moderately Disagree	2
		Strongly disagree	1
12	Using the LHIMS enhanced my effectiveness in healthcare delivery	Strongly Agree	7
		Moderately Agree	6
		Slightly Agree	5
		Neutral	4
		Slightly Disagree	3
		Moderately Disagree	2
		Strongly disagree	1
13	I intend to use LHIMS during my encounter with patient/client	Strongly Agree	7
		Moderately Agree	6
		Slightly Agree	5
		Neutral	4
		Slightly Disagree	3
		Moderately Disagree	2
		Strongly disagree	1
14	I believe it is a good idea to use LHIMS for healthcare delivery	Strongly Agree	7
		Moderately Agree	6
		Slightly Agree	5
		Neutral	4
		Slightly Disagree	3
		Moderately Disagree	2
		Strongly disagree	1
15	I intend to use the LHIMS as often as possible	Strongly Agree	7
		Moderately Agree	6
		Slightly Agree	5
		Neutral	4
		Slightly Disagree	3
		Moderately Disagree	2
		Strongly disagree	1
16	Learning to use the LHIMS interface was easy for me	Strongly Agree	7
		Moderately Agree	6
		Slightly Agree	5
		Neutral	4
		Slightly Disagree	3

Survey Variables and Codes			
		Moderately Disagree	2
		Strongly disagree	1
17	Using the LHIMS increased my productivity in my Work	Strongly Agree	7
		Moderately Agree	6
		Slightly Agree	5
		Neutral	4
		Slightly Disagree	3
		Moderately Disagree	2
		Strongly disagree	1
18	The LHIMS user interface was clear and understandable	Strongly Agree	7
		Moderately Agree	6
		Slightly Agree	5
		Neutral	4
		Slightly Disagree	3
		Moderately Disagree	2
		Strongly disagree	1
19	Using the LHIMS enabled me to accomplish tasks more quickly	Strongly Agree	7
		Moderately Agree	6
		Slightly Agree	5
		Neutral	4
		Slightly Disagree	3
		Moderately Disagree	2
		Strongly disagree	1
20	It was easy for me to become skilful at using the LHIMS interface	Strongly Agree	7
		Moderately Agree	6
		Slightly Agree	5
		Neutral	4
		Slightly Disagree	3
		Moderately Disagree	2
		Strongly disagree	1
21	Using the LHIMS system provided me with a lot of enjoyment	Strongly Agree	7
		Moderately Agree	6
		Slightly Agree	5
		Neutral	4
		Slightly Disagree	3
		Moderately Disagree	2
		Strongly disagree	1
22	I plan to use the LHIMS in the future	Strongly Agree	7
		Moderately Agree	6
		Slightly Agree	5
		Neutral	4
		Slightly Disagree	3
		Moderately Disagree	2
		Strongly disagree	1
23	I expect my use of LHIMS to continue in the future	Strongly Agree	7
		Moderately Agree	6
		Slightly Agree	5

**Survey Variables and Codes**			
		Neutral	4
		Slightly Disagree	3
		Moderately Disagree	2
		Strongly disagree	1
24	I found using the LHIMS useful	Strongly Agree	7
		Moderately Agree	6
		Slightly Agree	5
		Neutral	4
		Slightly Disagree	3
		Moderately Disagree	2
		Strongly disagree	1
25	Overall, I enjoyed using the LHIMS	Strongly Agree	7
		Moderately Agree	6
		Slightly Agree	5
		Neutral	4
		Slightly Disagree	3
		Moderately Disagree	2
		Strongly disagree	1
26	I found the LHIMS interface to be flexible to interact with	Strongly Agree	7
		Moderately Agree	6
		Slightly Agree	5
		Neutral	4
		Slightly Disagree	3
		Moderately Disagree	2
		Strongly disagree	1
